# A Rare Presentation of Systemic Lupus Erythematosus (SLE) With Hemoptysis and Hematuria: A Case Report

**DOI:** 10.7759/cureus.61161

**Published:** 2024-05-27

**Authors:** Gurjot Singh, Priya Antil, Kanishka Goswami, Tanya Ratnani, Piyush Puri

**Affiliations:** 1 Internal Medicine, Springfield Memorial Hospital, Springfield, USA

**Keywords:** genetics, hemodialysis, systemic steroids, pulmonary critical care, good pasture syndrome, diffuse alveolar hemorrhage, hemoptysis, hematuria, sle and lupus nephritis, sle

## Abstract

Systemic lupus erythematosus (SLE) is an autoimmune disease characterized by type II and type III hypersensitivity reactions that affect multiple organs, including the joints, heart, lungs, brain, skin, and kidneys. Patients with SLE can experience a range of symptoms, ranging from fever and joint pain to a distinctive butterfly facial rash. Severe complications may encompass conditions such as diffuse alveolar hemorrhage (DAH), pulmonary hypertension, and lupus nephritis, among others. Among them, DAH, a critical pulmonary complication in SLE, involves bleeding from interstitial capillaries and alveoli due to immune complex damage. This case report describes a patient who was initially misdiagnosed but later confirmed to have SLE. The patient presented with persistent symptoms, including cough, dyspnea, and fever, over two weeks and subsequently developed hematuria and hemoptysis within the last two days. The progression of symptoms led to an acute exacerbation, resulting in her admission to the emergency department. Subsequent evaluations confirmed the diagnosis of lupus nephritis and DAH. This case highlights the importance of considering SLE in the differential diagnosis of unexplained systemic symptoms and underscores the urgent need for medical intervention in DAH to substantially reduce mortality.

## Introduction

Systemic lupus erythematosus (SLE) is a chronic autoimmune disorder in which the immune system attacks the body’s own tissues, causing pervasive inflammation and damage in various organs, including the joints, skin, brain, lungs, kidneys, and blood vessels. The manifestations of SLE vary from mild to severe, affecting each patient uniquely [1].

In the United States, estimates of SLE prevalence have historically varied, with figures ranging from 24 to 150 cases per 100,000 people. However, recent analyses using data from US registries suggest a more precise prevalence rate of approximately 72.8 per 100,000 person-years [2-4].

A significant and serious complication of SLE is lupus nephritis, which often arises from a type III hypersensitivity reaction. This condition involves the accumulation of immune complexes (ICs) in various parts of the kidney, such as the mesangium and areas around the glomerular basement membrane (GBM). These deposits initiate an inflammatory response marked by complement activation and the recruitment of neutrophils and other immune cells. Both autoimmune mechanisms and genetic predispositions are key factors in the development of lupus nephritis [1,5,6].

Diffuse alveolar hemorrhage (DAH) represents a serious clinical syndrome linked to SLE, characterized by bleeding into the alveolar spaces as a result of disruption in the alveolar-capillary basement membrane. Symptoms of DAH include dyspnea, cough, hemoptysis, hypoxia, and usually a fever. Abnormal chest X-rays typically show bilateral alveolar infiltrates. High mortality rates of 70-92% have been reported in SLE patients who develop DAH [7]. Factors associated with poorer clinical outcomes include advanced age, long-standing disease, massive hemoptysis, secondary infections, thrombocytopenia, and the need for plasmapheresis. Improved survival rates are likely due to earlier diagnosis, more aggressive treatment, enhanced supportive care, early use of corticosteroids, and better ventilator management.

In this instance, initially, the clinical presentation raised suspicion of Goodpasture syndrome (GPS). However, this was ruled out after a renal biopsy revealed the absence of IgG antibodies against the basement membrane and instead showed mesangial proliferation. Further rheumatological evaluation identified a highly positive antinuclear antibody (ANA) titer (>1:2560), elevated anti-dsDNA (43 IU/mL), and positive anti-Smith antibodies, with negative anti-GBM antibodies. Following a thorough diagnostic workup, the patient was diagnosed with SLE.

The prompt administration of high-dose corticosteroids in conjunction with plasmapheresis played a crucial role in the successful treatment of a patient experiencing severe complications of SLE, specifically lupus nephritis and DAH.

## Case presentation

A 20-year-old woman without any chronic medical history was brought to the emergency department unconscious. The patient had presented with a persistent cough, dyspnea, and fever over the past two weeks and had recently developed gross hematuria and hemoptysis over the last two days. These symptoms had progressively worsened, culminating in her admission to the emergency department. Upon examination, she had a Glasgow Coma Scale score of 8/15 (E2, V2, M4) and exhibited widespread edema. Her vital signs included a heart rate of 112 bpm, blood pressure of 90/70 mmHg, a temperature of 98.5 °F (36.9 °C), and an oxygen saturation of 60% on room air, necessitating immediate intubation and mechanical ventilation.

Laboratory findings indicated anemia with a hemoglobin level of 6.45 g/dL and a mean corpuscular volume of 78.4 fl. The white blood cell count was 4.4 × 10^9^/L, consisting of 70% neutrophils, 20% lymphocytes, and 8% monocytes, while the platelet count was 179 × 10^9^/L. Renal function tests showed a creatinine level of 1.5 mg/dL, a blood urea nitrogen of 30 mg/dL, and a glomerular filtration rate (GFR) of 54% (estimated GFR of 51%). Liver function tests revealed alanine aminotransferase at 38 U/L, aspartate aminotransferase at 41 U/L, total bilirubin at 0.8 mg/dL, direct bilirubin at 0.2 mg/dL, and albumin at 2.5 g/dL. Electrolytes were within normal ranges, with sodium at 140 mEq/L and potassium at 4.9 mEq/L. Additional lab results showed a lactate dehydrogenase of 500 U/L, d-dimer at 198 ng/mL, fibrinogen at 400 mg/dL, and complement levels of C3 at 95 mg/dL and C4 at 20 mg/dL. Inflammatory markers were elevated, with CRP at 40 mg/dL and the erythrocyte sedimentation rate at 30 mm/hr. Other lab reports, including viral serology and coagulation parameters like PT/INR, were normal (Tables 1-3). The patient was started on a whole blood transfusion, low-dose corticosteroids, and supportive care.

**Table 1:** Hematology lab reports

Hematology	Results	Reference range
White blood cell count (1000/cumm)	4.4	12-15
Platelet count (1000/cumm)	179	150-450
Red blood cell count (million/uL)	3.89	4.5-5.1
Hemoglobin (g/dL)	6.45	12-14
Mean corpuscular volume (fL)	78.4	80-100
Mean corpuscular hemoglobin (pg)	26.5	27.5-33.2
Mean corpuscular hemoglobin concentration (gm/dL)	31.4	33.4-35.5
Neutrophils (%)	70	40-80
Lymphocytes (%)	20	20-40
Eosinophils (%)	2	1-6
Monocytes (%)	8	2-10
Basophils (%)	0	0-1
Erythrocyte sedimentation rate (mm/hr)	30	0-20

**Table 2:** Biochemistry lab reports

Biochemistry tests	Results	Reference range
Blood urea nitrogen (mg/dL)	30	15-40
Creatinine (mg/dL)	1.5	0.5-1.3
Glomerular filtration rate (mL/min/1.73 sq mm)	54	90-120
Total bilirubin (mg/dL)	0.8	0.2-1.0
Direct bilirubin (mg/dL)	0.2	<0.2
Indirect bilirubin (mg/dL)	0.6	0.2-0.8
Aspartate aminotransferase (U/L)	41	15-37
Alanine aminotransferase (U/L)	38	14-59
Alkaline phosphatase (U/L)	91	<98
Lactate dehydrogenase (U/L)	500	<250
Total protein (gm/dL)	5.9	6.4-8.3
Albumin (gm/dL)	2.5	3.2-4.5
Sodium (mEq/L)	140	135-145
Potassium (mEq/L)	4.9	3.5-5.0

**Table 3:** Miscellaneous lab reports

Miscellaneous	Results	Reference range
Antinuclear antibody	>1:2560	<1:40
Anti-dsDNA (IU/mL)	43	0-25
C3 (mg/dL)	95	90-150
C4 (mg/dL)	20	15-45
CRP (mg/dL)	40	<0.3

**Figure 1:**
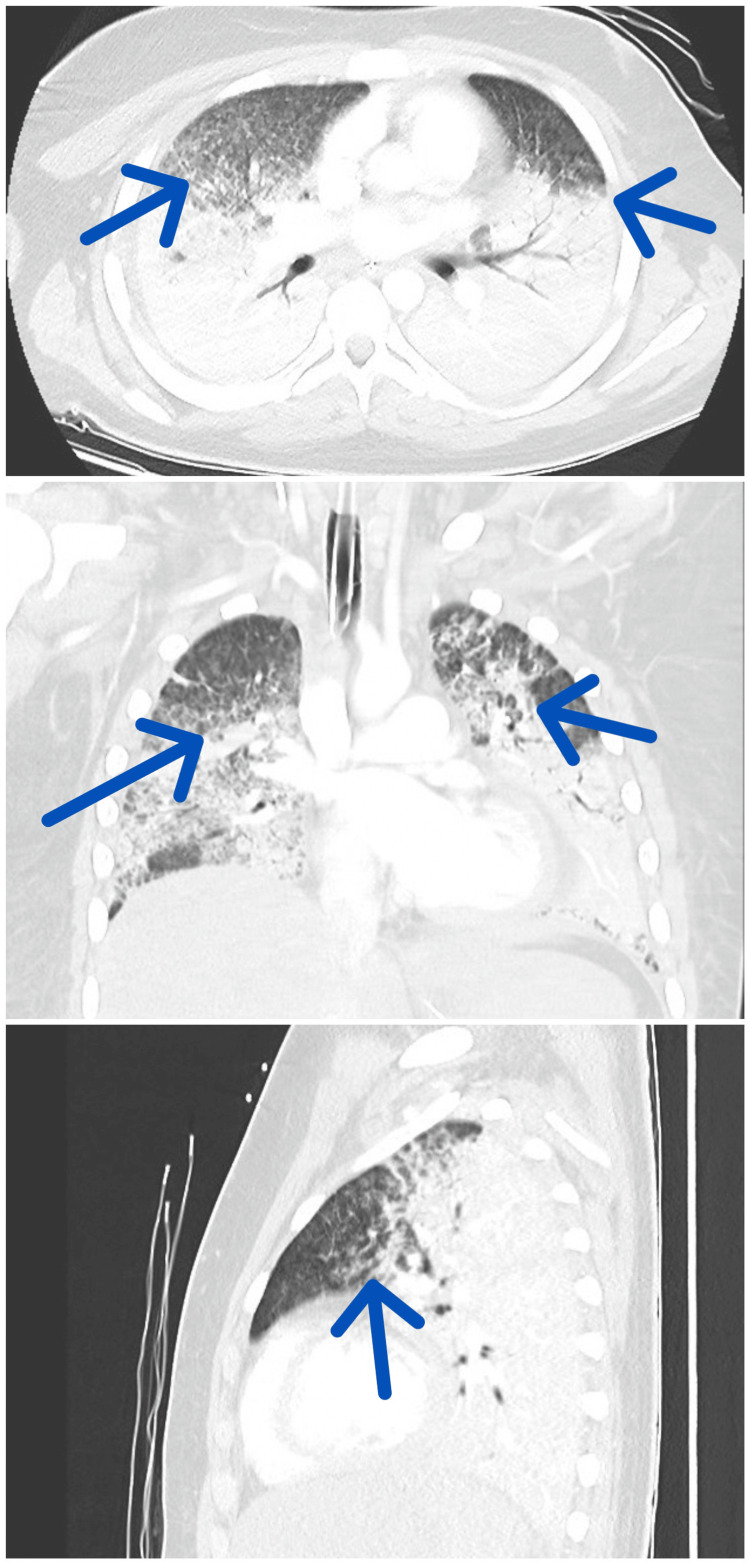
CT scan images suggestive of DAH (from top to bottom: axial, coronal, and sagittal views of the lung field) DAH, diffuse alveolar hemorrhage

Radiological evaluation included an MRI of the brain, which was unremarkable in determining the cause of unconsciousness. In contrast, a chest CT scan revealed DAH, indicated by widespread bleeding within the alveolar spaces (Figure 1).

**Figure 2:**
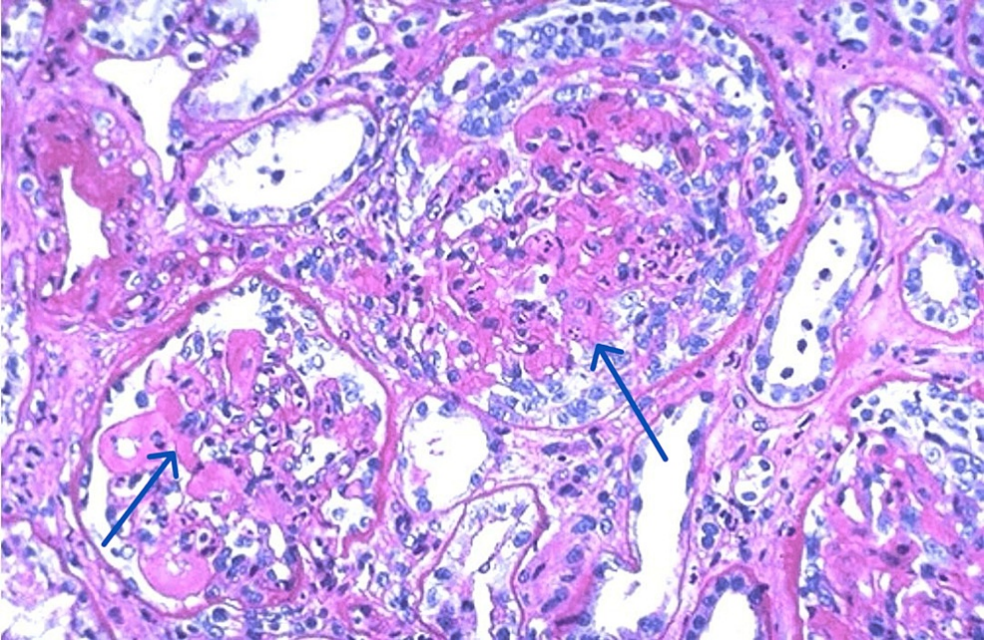
Kidney biopsy showing mesangial proliferation

Urinalysis showed red blood cell casts and proteinuria of 2+. Initially, the clinical presentation raised suspicions of GPS, but these were refuted following a renal biopsy, which was conducted after two days of hospitalization. The biopsy showed no IgG antibodies targeting the basement membrane. Instead, histologic examination revealed mesangial proliferation (Figure 2).

Immunofluorescence studies were positive for mesangial IgG, IgA, IgM, C3, and C1q. Electron microscopy confirmed the presence of subepithelial electron-dense deposits with segmental effacement of foot processes, consistent with mesangial proliferative lupus nephritis.

Further rheumatological evaluation revealed a highly positive ANA titer (>1:2560), anti-dsDNA (43 IU/mL), and anti-Smith antibodies, with negative anti-GBM. Tests like c-ANCA, p-ANCA, and APLA were negative.

Given the holistic history, including a family history of SLE in the sister, and the comprehensive diagnostic workup, the patient was diagnosed with SLE. The patient was started on high-dose steroids and plasmapheresis (a total of four plasma exchange cycles on alternate days, with one plasma volume exchanged during each cycle), leading to significant clinical improvement. The patient was eventually extubated, and her symptoms began to subside. The patient was later referred to rheumatology and started on maintenance therapy (with prednisolone and cyclophosphamide) for SLE.

## Discussion

SLE is a complex autoimmune disease with a wide range of clinical manifestations, including cutaneous lesions, chronic fatigue, arthritis, glomerulonephritis, and involvement of the neurological and cardiovascular systems. These symptoms may lead to severe complications, including strokes and other pulmonary issues, potentially resulting in premature death. SLE has a prevalence of 20-70 individuals per 100,000 and occurs six to 10 times more frequently in women than in men [2-4]. The pathogenesis of SLE is multifactorial, with both environmental and genetic contributors [7].

A distinguishing feature of SLE is impaired phagocytosis and clearance of apoptotic cells, leading to the accumulation of cell debris derived from the nucleus, cytosol, and membranes. This accumulation activates autoreactive B and T lymphocytes, resulting in the production of autoantibodies. These autoantibodies form ICs with nuclear and cytosolic antigens that circulate in the bloodstream, contributing substantially to the disease’s pathology. These ICs activate the classical pathway of the complement system, triggering inflammation in organs like the kidneys. Chronic complement activation results in complement depletion, further reducing the efficiency of phagocytic clearance of cell debris [6].

Involvement of the respiratory tract is observed in 50-70% of SLE patients and manifests as pleuritis, infiltrative pneumonia, bronchiolitis obliterans, and muscular and diaphragmatic dysfunction. Vascular complications associated with this condition can encompass a range of serious abnormalities, including pulmonary arterial hypertension, which involves elevated blood pressure within the pulmonary arteries; antiphospholipid syndrome, a disorder marked by excessive blood clotting; and DAH, characterized by widespread bleeding into the lung’s alveolar spaces [7-9]. Each of these complications presents significant clinical concerns requiring careful management and intervention.

DAH is a potential complication in SLE that may also arise from other systemic diseases such as small-vessel vasculitis (e.g., microscopic polyangiitis and granulomatosis with polyangiitis). Conditions like GPS, infections, increased left ventricular preload, and certain medications should also be considered in the differential diagnosis. Due to the high mortality (70-92%) associated with DAH, early and targeted diagnostic evaluations are crucial, as prompt, aggressive treatment can be lifesaving [7]. The importance of a precise diagnostic approach is emphasized by the rarity and severity of DAH in SLE, highlighting the need for clinical vigilance and expertise in managing these patients effectively.

The management of DAH primarily remains empirical, reflecting its status as a critical life-threatening emergency and the absence of established specific therapies. The prompt administration of high-dose corticosteroids may prove advantageous, although the overall mortality rate associated with DAH continues to be substantial. While plasmapheresis is recommended, its effectiveness is not as thoroughly documented as it is in other autoimmune conditions, such as GPS or specific vasculitis-associated pulmonary hemorrhages. Remarkably, in the case described, the early administration of high-dose corticosteroids combined with plasmapheresis was instrumental in saving the patient’s life. Consequently, our case report suggests that plasmapheresis may also be beneficial in managing severe complications like DAH and lupus nephritis in patients with SLE, as evidenced by the positive outcome in this instance.

## Conclusions

This case exemplifies the diagnostic complexity and clinical severity of SLE, particularly when presenting with acute symptoms such as hematuria and hemoptysis. Initially mistaken for GPS due to the presence of alveolar hemorrhage and renal involvement, the correct diagnosis was established through comprehensive serological and histological analyses, which confirmed SLE with lupus nephritis and DAH. This case underscores the critical need for a thorough differential diagnosis process in autoimmune diseases, where overlapping clinical features can obscure the underlying disorder. The successful identification and treatment of this patient’s condition demonstrates the effectiveness of integrating clinical findings with laboratory and histological data to manage complex cases of SLE effectively.
